# Supplementary material for: Neryl acetate, the major component of Corsican Helichrysum italicum essential oil, mediates its biological activities on skin barrier
Source: PLoS One. 2023 Mar 3;18(3):e0268384. doi: 10.1371/journal.pone.0268384 (PMC9983847; doi:10.1371/journal.pone.0268384)
Supplement: Supplementary file 1 [file pone.0268384.s001.docx]

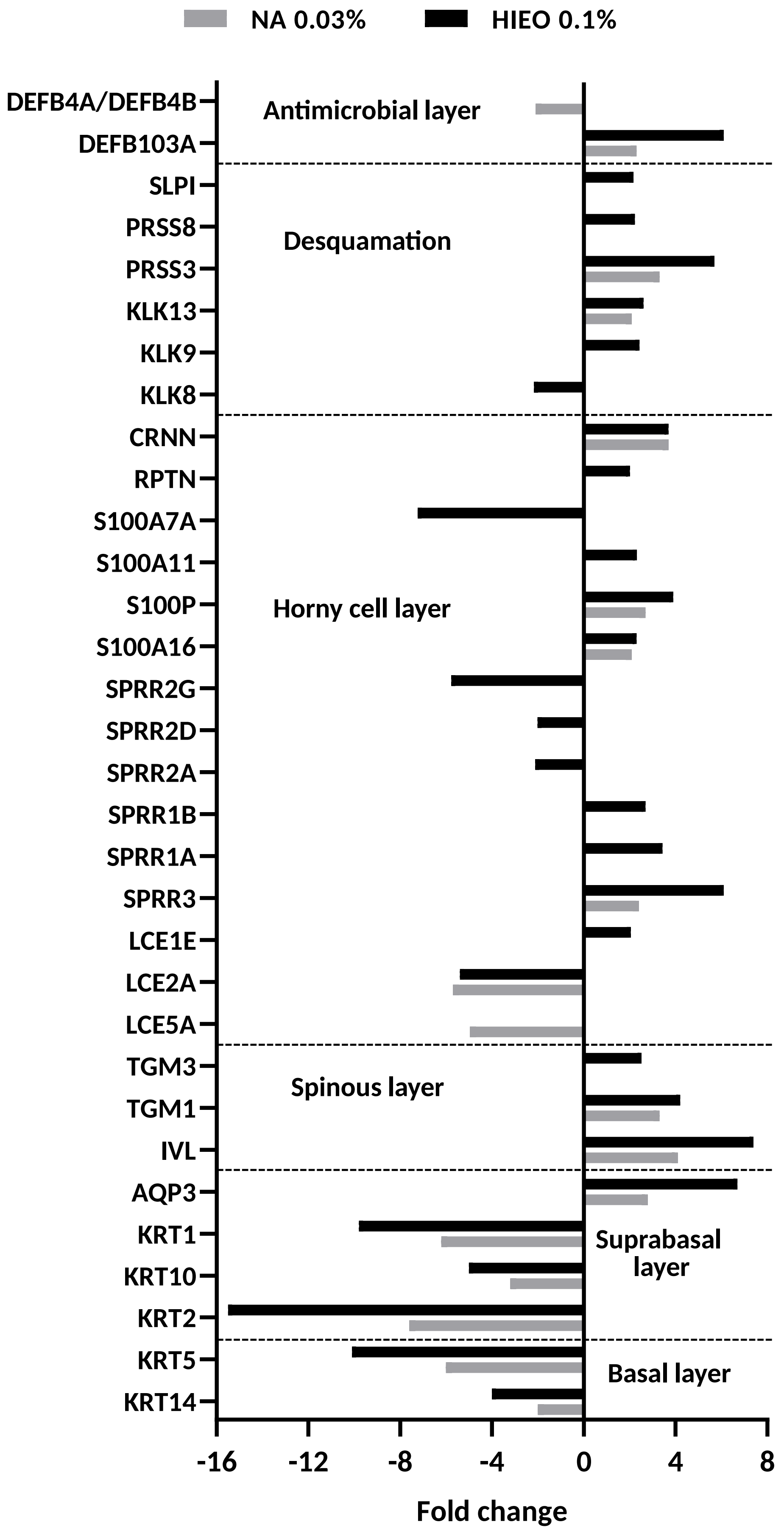
NA 0.03%
HIEO 0.1%
Antimicrobial layer
Desquamation
Horny cell layer
Spinous layer
Suprabasal layer
Basal layer
DEFB4A/DEFB4B
DEFB103A
SLPI
PRSS8
PRSS3
KLK13
KLK9
KLK8
CRNN
RPTN
S100A7A
S100A11
S100P
S100A16
SPRR2G
SPRR2D
SPRR2A
SPRR1B
SPRR1A
SPRR3
LCE1E
LCE2A
LCE5A
TGM3
TGM1
IVL
AQP3
KRT1
KRT10
KRT2
KRT5
KRT14
-16
-12
-8
-4
0
4
8
Fold change
