# Supplementary material for: Neryl acetate, the major component of Corsican Helichrysum italicum essential oil, mediates its biological activities on skin barrier
Source: PLoS One. 2023 Mar 3;18(3):e0268384. doi: 10.1371/journal.pone.0268384 (PMC9983847; doi:10.1371/journal.pone.0268384)
Supplement: Supplementary file 2 [file pone.0268384.s002.docx]

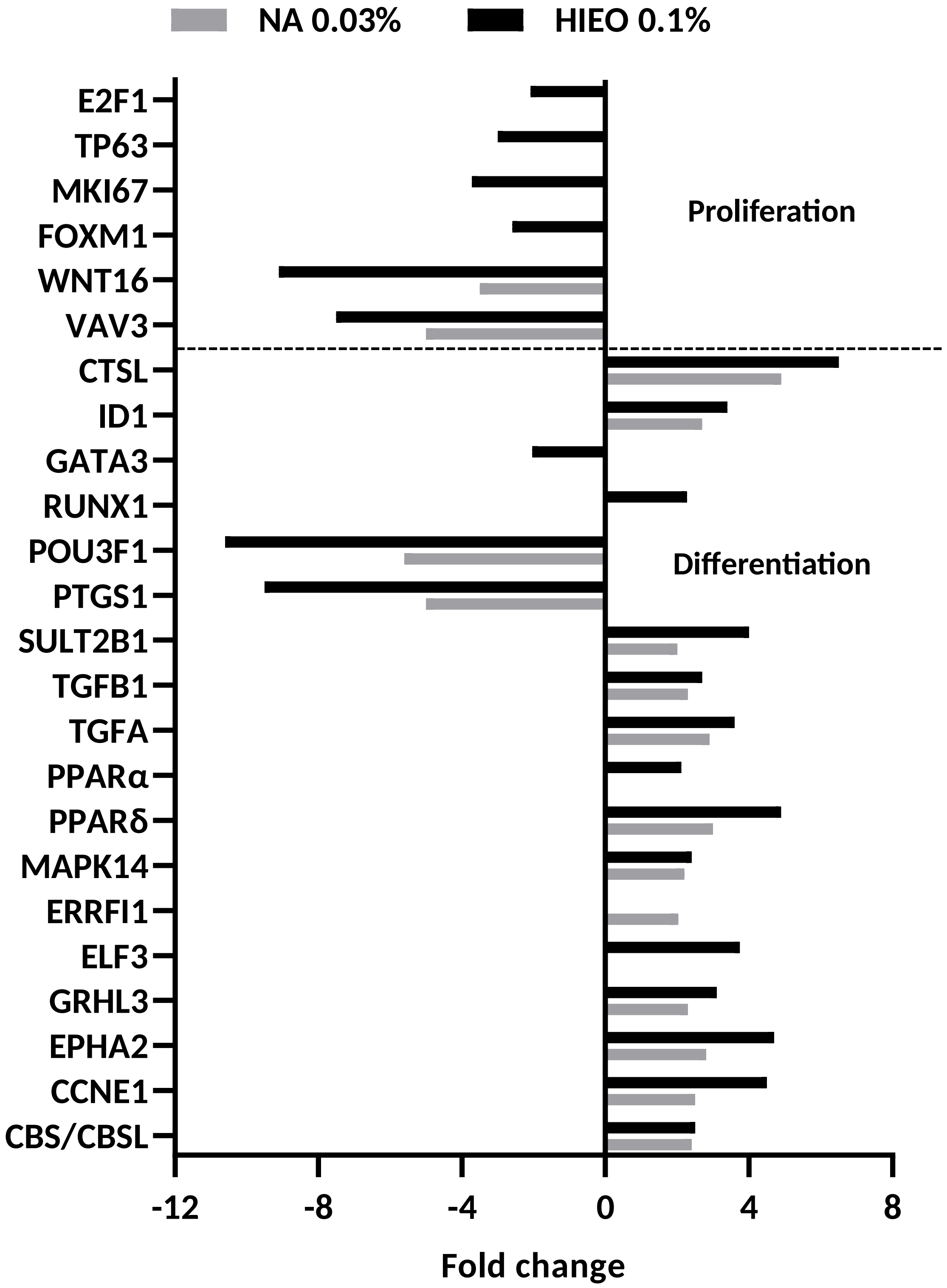
NA 0.03%
HIEO 0.1%
E2F1
TP63
MKI67
FOXM1
WNT16
VAV3
CTSL
ID1
GATA3
RUNX1
POU3F1
PTGS1
SULT2B1
TGFB1
TGFA
PPARα
PPARδ
MAPK14
ERRFI1
ELF3
GRHL3
EPHA2
CCNE1
CBS/CBSL
Proliferation
Differentiation
-12
-8
-4
0
4
8
Fold change
