# Supplementary material for: Neryl acetate, the major component of Corsican Helichrysum italicum essential oil, mediates its biological activities on skin barrier
Source: PLoS One. 2023 Mar 3;18(3):e0268384. doi: 10.1371/journal.pone.0268384 (PMC9983847; doi:10.1371/journal.pone.0268384)
Supplement: Supplementary file 3 [file pone.0268384.s003.docx]

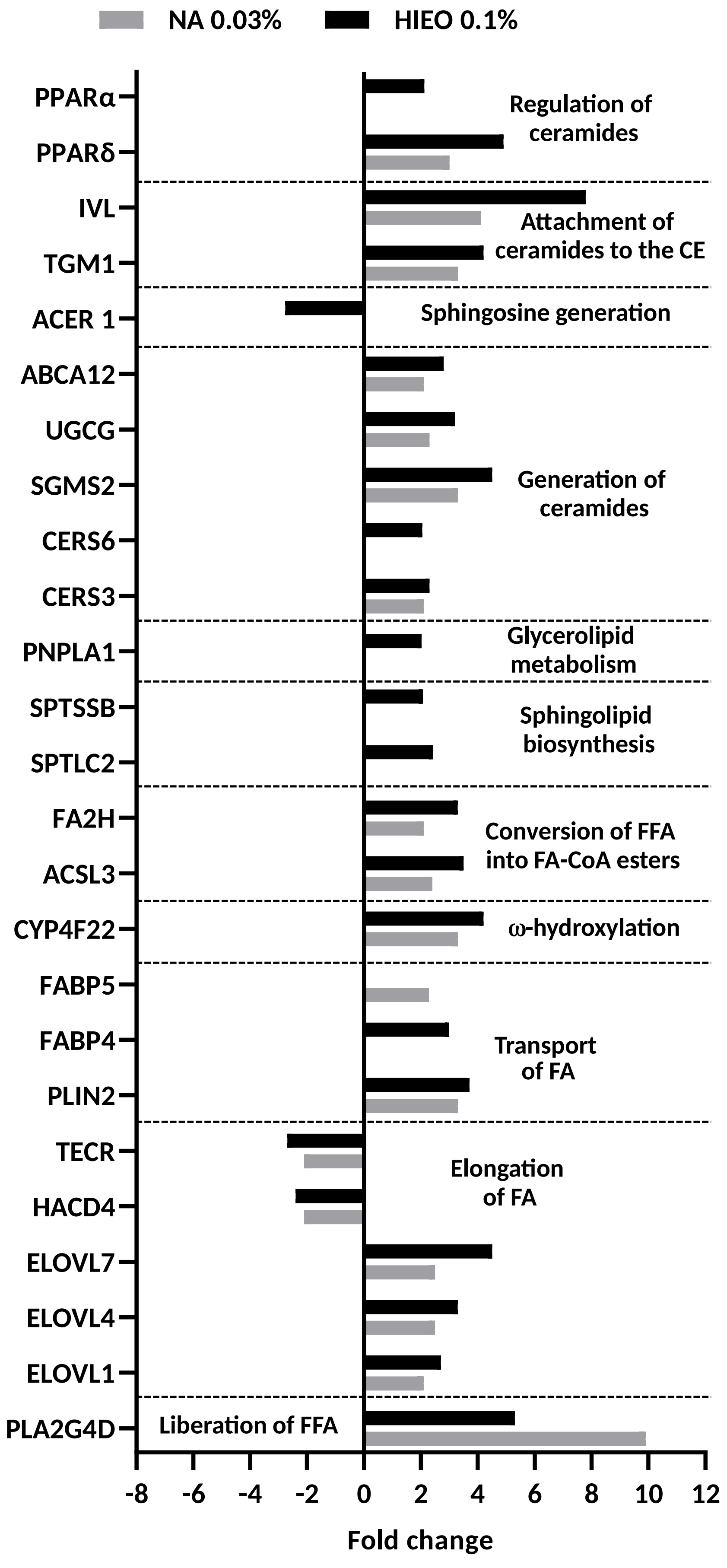
NA 0.03%
HIEO 0.1%
PPARα
PPARδ
IVL
TGM1
ACER 1
ABCA12
UGCG
SGMS2
CERS6
CERS3
PNPLA1
SPTSSB
SPTLC2
FA2H
ACSL3
CYP4F22
FABP5
FABP4
PLIN2
TECR
HACD4
ELOVL7
ELOVL4
ELOVL1
PLA2G4D
Regulation of ceramides
Attachment of ceramides to the CE
Sphingosine generation
Generation of ceramides
Glycerolipid metabolism
Sphingolipid biosynthesis
Conversion of FFA into FA-CoA esters
ω-hydroxylation
Transport of FA
Elongation of FA
Liberation of FFA
-8
-6
-4
-2
0
2
4
6
8
10
12
Fold change
