# Supplementary material for: Neryl acetate, the major component of Corsican Helichrysum italicum essential oil, mediates its biological activities on skin barrier
Source: PLoS One. 2023 Mar 3;18(3):e0268384. doi: 10.1371/journal.pone.0268384 (PMC9983847; doi:10.1371/journal.pone.0268384)
Supplement: Attachment — Submitted filename: Response to reviewers.docx [file pone.0268384.s010.docx]

PONE-D-22-12384R1

Neryl acetate, the major component of Corsican Helichrysum Italicum essential oil, mediates its biological activities on skin barrier

PLOS ONE

Dear Editor,

Please find my answer to the few points resting.

Kind regards,

Géraldine Lemaire

Reviewer #2: The authors significantly improved the manuscript and took into account the remarks.
There are still minor corrections to do:
- In the introduction, please take into account the difference between cornified envelope (CE) and corneocyte: line 62, about the major components of the stratum corneum, it would be more correct to write “four major components: the corneocyte, surrounded by the cornified envelope (CE) and the cornified lipid envelope (CLE), which are embedded in the intercellular lipid layers”. Similarly, line 68, write “fill the intracellular matrix of corneocytes” (instead of CE). Finally, line 74, write “the main reinforcement protein for the CE on its intracellular face” (instead of cytoplasmic face)
The sentences were corrected

- In the Materials and Methods section, lane 217: Table 2 instead of Table 1.
This was corrected

- In the Results section, line318: KRT2 is downregulated as KRT1 and KRT10, thus correct the text.
This was corrected

- In Fig5C: Please change “Tight junctions” by “intercellular junctions” (which include other junctions than TJs)
This was corrected in the figure 5C

- In the Results section, line 357: It’s better writing “involved in the formation of TAG and linoleic acid, and the metabolism of cholesterol”. Line 378: “involves TAG synthesis, linoleic acid synthesis and cholesterol metabolism”.
This was corrected

- There is a confusion between LIPN and PNPLA1. Line 360 (result section) and lines 495-496 (Discussion section): this is PNPLA1 and not LIPN which works in association with ABHD5 to synthesize omega-O-acylceramides. This has to be corrected. As I previously notified concerning LIPN, its function in the metabolism of epidermal lipids is not yet clearly known.

This was corrected as the following sentences: As show in Fig 7A, genes involved in the formation of TAG, linoleic acid, and the metabolism of cholesterol were regulated after 24 hours of NA and HIEO treatment. Indeed, *glycerol kinase* (*GK*) convert glycerol into glycerol 3 phosphate, the substrate of *glycerol-3-phosphate acyltransferase 3* (*GPAT3*) that catalyze the first step of TAG synthesis and *patatin like phospholipase domain containing 1* (*PNPLA1)* in association with his cofactor *abhydrolase domain containing 5* (*ABHD5*) is facilitate TAG lipolysis to produce linoleic acid. Among the various candidates that could hydrolyze TAG, the expression of *lipase family N* (LIPN) was increased by NA and HIEO.

And the sentence was deleted in the discussion as following: Since *PPARδ* stimulates keratinocyte differentiation and ceramide synthesis [42], it seems reasonable to consider the possible activation of *PPARδ* by NA content in HIEO, probably indirectly by the well-known PPARδ ligand linoleic acid.
